# Exploring the Therapeutic Potential of *Allium cepa* and *Allium sativum* Extracts: Current Strategies, Emerging Applications, and Sustainability Utilization

**DOI:** 10.3390/biology14081088

**Published:** 2025-08-20

**Authors:** Alaa S. Bedir, Razan S. Almasri, Yasmena O. Azar, Rana E. Elnady, Seham M. Al Raish

**Affiliations:** 1 Department of Nutrition, College of Medicine and Health Science, United Arab Emirates University, Al Ain 15551, United Arab Emirates; 201950078@uaeu.ac.ae (A.S.B.); 201950110@uaeu.ac.ae (R.S.A.); 2 Department of Pharmacology and Toxicology, Faculty of Pharmacy, Sinai University–Arish Branch, Arish 45511, Egypt; yasmena.osama@su.edu.eg; 3 Department of Pharmaceutics, Faculty of Pharmacy, Sinai University–Arish Branch, Arish 45511, Egypt; rana.essam@su.edu.eg; 4 Department of Biology, College of Science, United Arab Emirates University, Al Ain 15551, United Arab Emirates

**Keywords:** antibacterial effects, antidiabetic activity, cardioprotective properties, garlic, nanotechnology, onion

## Abstract

This review explores the therapeutic potential of *Allium cepa* (onion) and *Allium sativum* (garlic), focusing specifically on their antidiabetic, cardioprotective, and antibacterial effects. These widely used medicinal plants are rich in bioactive compounds that contribute to their broad spectrum of pharmacological activities. Based on over two decades of scientific literature, this review synthesizes findings from more than 20 years of research to highlight the efficacy of these plant extracts. Rising chronic disease rates and antimicrobial resistance have renewed interest in plant-derived therapies; in the UAE context, *Allium cepa* and *Allium sativum* are promising candidates for integrative, sustainable interventions. This review further elucidates the mechanisms through which those bioactive constituents exert therapeutic effects, current extraction strategies, and challenges in enhancing formulations for functional health applications. Additionally, it addresses their role in sustainable healthcare practices due to their renewable nature and minimal environmental impact compared to synthetic pharmaceuticals.

## Simple Summary

Onions and garlic are not only kitchen staples but also valuable natural remedies. This review looks at how extracts from these plants may help in preventing or managing some of today’s most pressing health problems, such as diabetes, heart disease, and bacterial infections. Both plants contain natural chemicals with protective effects that can support healthy blood sugar levels, improve heart health, and fight harmful microbes. By bringing together more than twenty years of scientific research, we explain how these plants work in the body and why they are attracting attention from scientists and healthcare providers. In the United Arab Emirates, where such health issues are growing, onions and garlic offer affordable and accessible options that also align with environmentally friendly healthcare practices. Because they are renewable and have a low impact on the environment, they represent an approach that benefits both people and the planet. This work also discusses modern methods to make these natural remedies more effective, paving the way for their use alongside conventional treatments in the future.

## 1. Introduction

Medicinal plants (MP) have traditionally been integral to both traditional and contemporary medicine globally, providing numerous therapeutic advantages. In recent years, there has been a significant increase in interest in plant-based therapies as individuals and researchers pursue alternative and complementary approaches for chronic health issues, especially metabolic disorders such as diabetes, cardiovascular diseases (CVDs), and infections resistant to antimicrobials [1,2], as there are deep relationships between the plants and microorganisms [3]. The United Arab Emirates (UAE), characterized by its distinctive desert vegetation and extensive history of traditional medicine, serves as a significant repository of medicinal plants that are both culturally relevant and pharmacologically advantageous [4].

The incidence of diabetes and CVD in the UAE is increasing, reflecting global patterns linked to lifestyle modifications and urbanization [5,6,7]. These trends heighten the need for effective, accessible interventions that complement standard care. The challenge of antimicrobial resistance is concurrently prompting an urgent quest for innovative solutions, rendering plant-derived antimicrobials a compelling research focus [8]. The native and adapted flora of the UAE, recognized for its resilience to extreme climatic conditions, contains bioactive compounds that may effectively address and alleviate these health issues [9].

The hyper-arid environment of the UAE accommodates a diverse array of MP. The utilization of MP is prevalent in the UAE, with potential adverse reactions in patients. In 2011, at a Nephrology clinic in Abu Dhabi, UAE, the Emirati population demonstrated strong faith and confidence in the use of MP, being well-acquainted with the therapeutic and flavoring attributes of various herbs [10,11]. Herbs are conventionally employed for the treatment and prevention of conditions such as abdominal pain, cephalalgia, diabetes, hypertension, rheumatism, and various other prevalent purposes, including immune enhancement, relaxation, and cosmetic applications [11,12]. The UAE possesses a significant variety of flora that have adapted to harsh and temperate environments and have medicinal properties [13].

This review synthesizes the existing scientific literature on MP in the UAE, emphasizing its established antidiabetic, cardiovascular, and antibacterial properties. Within this context, *Allium cepa* (onion) and *Allium sativum* (garlic) merit focused attention because their extracts have been repeatedly linked to antidiabetic, cardioprotective, and antibacterial effects. It aims to underscore the significance of these plants’ pharmacological profiles and potential therapeutic applications in tackling the UAE’s urgent health challenges. The review also addresses the difficulties in standardizing, preserving, and utilizing MP, which is essential for ensuring that these resources contribute to enduring health solutions [14,15].

This study enhances the global understanding of ethnopharmacology and promotes additional investigations of the UAE’s botanical assets. It could potentially result in the creation of effective, plant-derived therapeutic agents that conform to sustainable healthcare principles. By deepening global insights into ethnopharmacology, this review encourages further research into the UAE’s rich botanical resources. It holds the potential to pave the way for the development of effective plant-based therapeutic agents aligned with sustainable healthcare practices. Moreover, the current strategies used to formulate plant-based therapies to enhance their therapeutic effects are aligned with sustainable healthcare practices.

## 2. Methodology

### 2.1. Selection Criteria

#### 2.1.1. Data Collection

Between 1998 and 2025, a comprehensive review of the flora of the UAE was undertaken by compiling and critically analyzing a wide range of research sources, including reputable online platforms. This investigation focused primarily on MPs such as *Allium cepa* (*A. cepa*) and *Allium sativum* (*A. sativum*), which are well-documented for their antidiabetic, cardioprotective, and antibacterial properties. Botanical names and their synonyms were systematically collected, and an exhaustive literature search was conducted for each species, prioritizing plants native to or naturalized within the UAE and recognized for their medicinal applications. Digital resources, including Google Scholar and PubMed, were utilized to develop a targeted search strategy, combining each plant’s botanical name or synonym with relevant keywords to specifically identify studies on their antidiabetic, antimicrobial, and cardioprotective activities.

#### 2.1.2. Inclusion Criteria

The inclusion criteria were designed to capture studies relevant to the geographical context of the UAE, with a specific focus on *Allium cepa* (onion) and *Allium sativum* (garlic). These two species were selected due to their prevalence in the region and their well-documented therapeutic properties. Eligible studies investigated their antidiabetic, cardioprotective, or antimicrobial effects and were published in reputable, peer-reviewed journals within the past 20 years. The considered research types included experimental studies (both in vitro and in vivo), observational studies, ethnobotanical surveys, and comprehensive reviews. Preference was given to studies providing robust pharmacological data, including the identification of active compounds, the elucidation of mechanisms of action, and defined therapeutic applications. Only articles published in English or other languages with reliable English translations were accepted.

#### 2.1.3. Exclusion Criteria

Studies were excluded if they did not pertain to *Allium cepa* or *Allium sativum* of relevance to the UAE, or if they lacked a clear medicinal focus. Research from non-peer-reviewed sources, publications in languages other than English without dependable translations, and studies not addressing antidiabetic, cardioprotective, or antimicrobial effects were also omitted. This ensured that only high-quality, relevant evidence aligned with the objectives of this review was included.

## 3. Medicinal Plants in the UAE Overview

### 3.1. Taxonomy and Botanical Classification of Allium cepa and Allium sativum

The genus *Allium* L. comprises over 1100 recognized species, making it one of the largest genera in the family Amaryllidaceae and a taxonomically significant group within the order Asparagales [16]. These species are widely distributed across temperate regions of the Northern Hemisphere, with many cultivated globally for culinary and medicinal purposes [17]. The formal botanical classification of *Allium cepa* and *Allium sativum* is presented below [18]:

○ Kingdom: Plantae
○ Clade: Angiosperms
○ Clade: Monocots
○ Order: Asparagales
○ Family: Amaryllidaceae
○ Subfamily: Allioideae
○ Genus: *Allium* L.
○ Species:
  ▪ *Allium cepa* L. (common onion)
  ▪ *Allium sativum* L. (garlic)

*Allium cepa* is a biennial herbaceous plant that is typically cultivated annually and characterized by tunicate bulbs, hollow cylindrical leaves, and terminal umbels bearing white to purplish flowers [19]. *Allium sativum* is a bulbous perennial species propagated almost exclusively through cloves, producing flat leaves and compound bulbs composed of multiple bulblets. Both species are notable for their organosulfur compounds, which contribute to their characteristic aroma, flavor, and wide range of pharmacological activities [20]. Originating in Central and Southwestern Asia, these plants have been cultivated for millennia, and their enduring integration into human diets and traditional medicine systems underscores their economic, cultural, and therapeutic importance [19,20].

### 3.2. Allium cepa

*Allium cepa*, commonly referred to as onion, is an extensively cultivated and consumed biennial plant within the genus *Allium* of the Amaryllidaceae family. It is typically cultivated in fields from October to December [21]. It is rich in fiber and essential nutrients, including vitamins B6 and B9 (folic acid) and minerals such as magnesium, calcium, potassium, and phosphorus [22,23]. Beyond its nutritional value, *A. cepa* has been extensively researched for its health benefits, which include antimicrobial, anticancer, antidiabetic, antioxidant, antihypertensive, and antidepressant properties [22,24]. Additionally, it offers neuroprotective, anti-inflammatory, and cardioprotective effects, benefiting the digestive, circulatory, respiratory, cardiac, and immune systems [22,25].

Additionally, *A. cepa* is a rich source of quercetin, a potent antioxidant found in both free and conjugated forms. Quercetin provides a range of health benefits, including anti-inflammatory, anti-proliferative, anti-carcinogenic, anti-diabetic, and anti-viral activities, potentially offering protection against aging [26]. Additionally, *A. cepa* contains anthocyanins, which have gained attention due to the improved methods of extraction that enhance both their yield and stability [27].

*Allium cepa* waste, particularly peels, is valuable due to its high content of bioactive compounds like flavonoids, polyphenols, and quercetin. *A. cepa* peel extracts have demonstrated antimicrobial, neuroprotective, anti-cancer, and anti-hyperglycemic effects, as well as benefits against hypercholesterolemia, obesity, and erectile dysfunction, making them useful for biomedical and pharmaceutical applications [28]. With its diverse bioactive components, *A. cepa* shows great potential for therapeutic and industrial uses [29].

### 3.3. Allium sativum

*Allium sativum* (garlic), a widely used herbaceous perennial plant, is known for its culinary and medicinal applications across the globe [30,31]. Cultivated in various regions, including UAE nurseries [21], *A. sativum* is rich in bioactive sulfur compounds like allicin, alliin, and ajoene, as well as flavonoids such as quercetin [31,32]. Studies have shown that *A. sativum* possesses a wide range of therapeutic properties, including anticarcinogenic, antioxidant, antidiabetic, renoprotective, anti-atherosclerotic, antimicrobial, anti-obesity, and antihypertensive effects [33,34,35]. Its bioactive compounds are particularly responsible for modulating immune function, enhancing cytokine expression, and exhibiting antimicrobial, anti-inflammatory, and anticancer effects [34,36].

*Allium sativum* contains several sulfur-containing amino acids, notably S-allyl-L-cysteine, which is derived from the hydrolysis of γ-glutamyl-S-allyl-L-cysteine [28]. The plant’s composition varies with type and preparation method, containing both sulfur and non-sulfur compounds. These compounds are central to *A. sativum*’s health benefits, contributing to its antioxidant, anti-inflammatory, and immune-enhancing properties. Among the primary bioactive compounds are diallyl polysulfides, vinyldithiins, and diallyl sulfide, which are associated with their therapeutic effects, including anti-infective, antimicrobial, and anticancer properties [37]. These findings underscore *A. sativum*’s potential as a natural remedy for various health conditions.

## 4. Overview of Antidiabetic Properties of Medicinal Plants in the UAE

### 4.1. Allium cepa

The potential of *A. cepa* as a natural therapeutic agent for the management of diabetes and the complications that are associated with it are highlighted by the various mechanisms that it incorporates. Research evidence provides substantial support for the potential anti-diabetic effects of *A. cepa* and the bioactive compounds that it contains. *A. cepa* contains a wide variety of bioactive compounds, which contribute to their therapeutic potential, particularly in managing diabetes complications. The primary bioactive compound, quercetin, possesses properties that make it effective in the treatment of type 2 diabetes mellitus (T2DM) [26].

These properties include antioxidant, anti-inflammatory, hypoglycemic, and hypolipidemic effects. According to Koh et al. (2015) and Li et al. (2015), the anthocyanins present in red *A. cepa* play a significant part in the process of insulin secretion and protect pancreatic beta cells from oxidative stress caused by glucose [38,39]. Compounds that contain sulfur, such as S-methyl cysteine sulfoxide, have been shown to stimulate insulin secretion, regulate blood glucose levels, and normalize the activities of liver enzymes such as hexokinase, glucose 6-phosphatase, and 3-Hydroxy-3-Methylglutaryl-Coenzyme A. reductase [24]. On the other hand, S-allyl cysteine sulfoxide increases insulin production and secretion while lowering the amount of glucose absorbed from the diet. Another study found that chromium and allyl propyl disulfide contribute to a reduction in fasting blood sugar and insulin levels, while also improving glucose tolerance [24].

**Figure 1:**
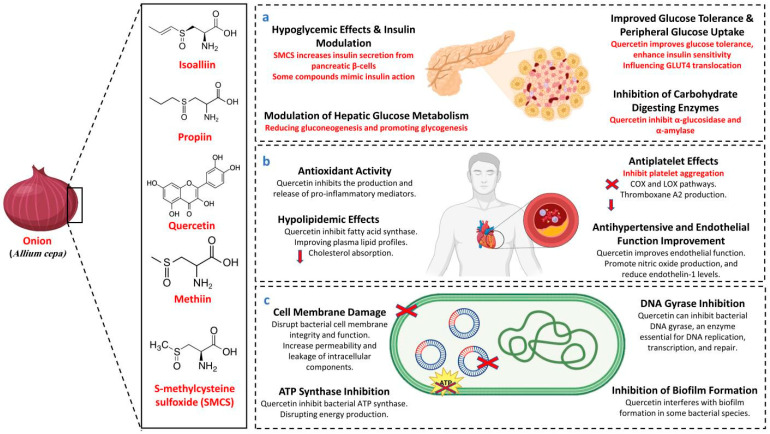
Mechanism of *A. cepa* as an (**a**) anti-diabetic, (**b**) cardioprotective, (**c**) anti-bacterial. GLUT4 (glucose transporter type 4), COX (cyclooxygenase), LOX (lipoxygenase).

In diabetic animal models, quercetin, a major flavonoid found in *A. cepa*, has been shown to reduce blood glucose levels, preserve the function of islet cells, and maintain the number of β-cells. These findings have been demonstrated through preclinical studies. Research conducted by Refat et al. (2021) and Zu et al. (2021) suggests that quercetin consumption may be advantageous in the prevention of diabetes mellitus as well as in the treatment of the disease [40,41]. The anti-diabetic effects of *A. cepa* have been well documented in traditional medicine and modern research [24,42]. The anti-diabetic mechanism of *A. cepa* is illustrated in Figure 1a.

### 4.2. Allium sativum

*Allium sativum* is rich in bioactive compounds that contribute to its anti-diabetic effects through diverse mechanisms. Sulfur-containing compounds such as allyl propyl disulfide, allicin, and S-allyl cysteine sulfoxide lower blood glucose levels by enhancing insulin secretion, improving insulin sensitivity, and preventing insulin inactivation by the liver [34,35]. Other sulfur compounds, like diallyl disulfide and diallyl trisulfide, have also demonstrated antidiabetic properties [43].

Numerous clinical trials have explored *A. sativum*’s impact on glycemic control in patients with T2DM. Studies have reported significant improvements in fasting blood glucose, glycated hemoglobin (HbA1c), and insulin sensitivity [44,45,46]. *A. sativum* supplementation has also shown potential to improve metabolic parameters like cholesterol and triglyceride levels, offering a complementary approach to conventional diabetes management [35]. In vivo, research has further supported *A. sativum*’s efficacy, with fresh *A. sativum* extract interventions improving insulin levels and lowering blood glucose and HbA1c in diabetic rats [47].

Specific *A. sativum* preparations, including allicin (200 mg/kg), aged *A. sativum* extract, and *A. sativum* oil, have demonstrated potent antidiabetic effects.

These include reductions in blood sugar comparable to standard medications, improved glucose tolerance, and enhanced kidney and liver function in diabetic animal models [48]. Additionally, *A. sativum*’s bioactive compounds, such as caffeic acid 3-glucoside and calenduloside E, have shown Dipeptidyl peptidase-IV inhibitory activity, aiding diabetes management [49]. Clinical trials in T2DM patients using *A. sativum* powder supplementation have revealed reductions in fasting plasma glucose levels within six weeks [47].

**Figure 2:**
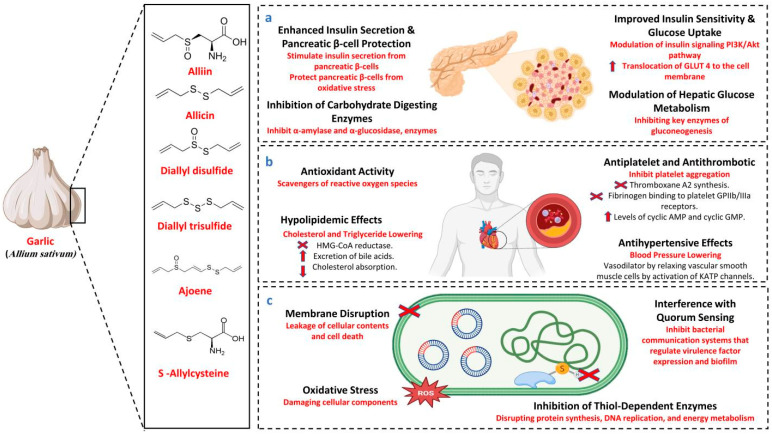
Mechanism of *A. sativum* as (**a**) anti-diabetic, (**b**) cardioprotective, (**c**) anti-bacterial. GLUT4 (glucose transporter type 4), HMG-COA (3-hydroxy-3-methylglutaryl coenzyme A), AMP (activated protein kinase), GMP (activated protein kinase), KATP (ATP-sensitive potassium) channel).

Emerging research highlights innovative uses of *A. sativum*, including its role in synthesizing silver nanoparticles (AgNPs). Biosynthesized from *A. sativum* extract, these AgNPs have demonstrated potent antioxidant and antidiabetic activity, further enhancing *A. sativum*’s therapeutic potential [50]. With its multifaceted mechanisms and extensive clinical support, *A. sativum* remains a promising complementary agent in diabetes management, offering potential benefits for glycemic control and overall metabolic health. The anti-diabetic mechanism of *A. sativum* is illustrated in Figure 2a.

## 5. Overview of Cardioprotective Properties of Medicinal Plants in the UAE

### 5.1. Allium cepa

*Allium cepa*, particularly rich in the flavonoid quercetin, has shown promising cardiovascular benefits. Studies on quercetin-rich *A. cepa* peel extract (OPE) in Sprague Dawley rats demonstrated reduced blood triglyceride and glucose levels and delayed arterial thrombosis, primarily through the downregulation of the Mitogen-activated protein kinase signaling pathway. This included the inhibition of extracellular signal-regulated kinase and c-Jun N-terminal kinase phosphorylation, reducing tissue factor expression in endothelial cells, and suggesting potent antithrombotic effects [28]. Onion extracts and certain chemicals, such as quercetin and onion thiosulfinates, have been proven to stop platelets from sticking together. This may be because they stop the cyclooxygenase (COX) and lipoxygenase (LOX) pathways, which lowers the formation of thromboxane A2 [51].

In human studies, flavonol-rich *A. cepa* extracts have been shown to lower blood pressure and improve endothelial function, especially in smokers and overweight individuals. Daily supplementation with 100 mg of quercetin enhanced postprandial flow-mediated dilation and endothelial progenitor cell counts, although not all *A. cepa* fractions, such as *A. cepa* skin extract, yielded cardioprotective effects [52].

Red *A. cepa*, a rich source of quercetin, is traditionally valued for its immunostimulant, antioxidant, and cardioprotective properties [34]. Quercetin’s potent antioxidant and anti-inflammatory actions contribute to cardiovascular health, including its role in lowering blood pressure and preventing atherosclerosis [53]. Experimental findings suggest that *A. cepa*’s cardioprotective effects depend on dosage, with aqueous extracts at 400 mg/kg showing protection against myocardial injury and higher doses lacking significant effects [54]. Additionally, quercetin from *A. cepa* has been shown to act as a complementary antioxidant in atherosclerotic regions, reinforcing its role in cardiovascular health [55]. Quercetin stops the creation and release of pro-inflammatory mediators, including histamine, leukotrienes, prostaglandins, and cytokines, by blocking enzymes like COX and LOX and changing signaling pathways like NF-κB [56]. The cardioprotective mechanism of *A. cepa* is illustrated in Figure 1b.

### 5.2. Allium sativum

*Allium sativum* has demonstrated potent antihypertensive properties through multiple mechanisms. Bioactive compounds like gamma-glutamylcysteine inhibit angiotensin-converting enzyme (ACE), contributing to blood pressure reduction [34]. Experimental and clinical studies have shown that *A. sativum* extracts increase plasma fibrinolytic activity and reduce thromboxane B2 and prostaglandin E2 levels in hypertensive models, supporting its role in managing hypertension [34]. Additionally, AGE has been shown to enhance nitric oxide synthesis and endothelial-dependent vasodilation, with L-arginine playing a critical role in these effects [57]. These findings establish *A. sativum* as a valuable adjunct in hypertension therapy.

*Allium sativum* exhibits cardioprotective properties through anti-inflammatory, antioxidant, and anti-apoptotic mechanisms. Allicin, a key bioactive compound, shows potential in treating CVDs such as atherosclerosis, hypertension, and heart failure, though challenges like poor bioavailability and odor remain [58]. Hydroalcoholic *A. sativum* extract further supports heart health by targeting oxidative stress and inflammation through phenolic compounds like gallic acid, alliin, and quercetin-3-galactoside, which interact with inflammatory biomarkers and enzymes like Cyclooxygenase-2 [59]. Moreover, *A. sativum* supplements reduce cardiac and mitochondrial dysfunction in insulin-resistant and obesity models, highlighting their broad cardiovascular benefits [57].

Clinical evidence supports *A. sativum*’s role in reducing cardiovascular risk factors, including blood pressure, low-density lipoprotein cholesterol, and platelet aggregation. Long-term supplementation has been linked to a reduced 10-year risk of coronary heart disease and myocardial infarction [60]. A study demonstrated that *A. sativum* powder pills slowed carotid atherosclerosis progression, underscoring their preventive potential. Additionally, AGE significantly improves vascular function and reduces systolic blood pressure, while time-released tablets like *allicor* show sustained anti-hypertensive effects [61]. These studies collectively affirm *A. sativum*’s efficacy in cardiovascular disease prevention and management. The cardioprotective mechanism of *A. sativum* is illustrated in Figure 2b.

## 6. Overview of Antibacterial Properties of Medicinal Plants in the UAE

### 6.1. Allium cepa

*Allium cepa* is recognized for its effectiveness in treating infectious diseases, with studies showing that its essential oil, derived from red, green, and yellow *A. cepa*, exhibits significant antimicrobial activity against pathogens such as *Salmonella enteritidis*, *Fusarium oxysporum*, *Penicillium cyclopium*, *Staphylococcus aureus* (*S. aureus*), and *Aspergillus niger* [22]. Onion thiosulfinates can react with thiol groups in bacterial enzymes, leading to their inactivation and disruption of cellular functions [62]. Quercetin extracted from *A. cepa* peels enhances these antimicrobial effects by disrupting energy metabolism, membrane functions, and nucleic acid biosynthesis, particularly against S. aureus [28]. *A. cepa* peel-derived nanoparticles also promise applications in medicine, cosmetics, food packaging, and pharmaceuticals, providing antibacterial and anti-aging benefits [28].

Six *A. cepa* varieties, red-skinned (*Happyhong*, *Unijinara*), yellow-skinned (*Marusino330*, *Sinsunhwang*), and white-skinned (*Mokpo24ho*, *Mokpo21ho*), were studied for bioactive compounds like flavonoids, phenolics, antioxidants, and antimicrobial activity over storage durations. After three months, the total phenolics, flavonoids, and antioxidant activities increased significantly in all varieties. Red *A. cepa* showed the most potent antimicrobial and antibiofilm activity, moderate effects in yellow *A. cepa*, and minimal activity in white *A. cepa* [63]. Furthermore, *A. cepa* juice and fresh bulb extract effectively reduced *C. difficile* populations and toxin production within 48 h, demonstrating the potential to mitigate infections [64].

The combined extract of *Nigella sativa*, *Syzygium aromaticum*, and *A. cepa* exhibited antimicrobial activity against *S. aureus*, *Candida albicans*, *Streptococcus mutans*, and *Enterococcus faecalis*, with inhibition zones ranging from 16 and 22 mm at 100 μL concentration, though less potent than control antibiotics [65]. Additionally, due to its phenolic and protein compounds, *A. cepa* peel extract proved highly effective against *Bacillus cereus*, *Escherichia coli* (*E. coli*), *S. aureus*, and others. Combined with barbecue flavor, *A. cepa* peel extract significantly reduced pathogens like *Listeria monocytogenes* in irradiated pork, ensuring microbial safety during storage [2,66]. The antibacterial mechanism of *A. cepa* is shown in Figure 1c.

### 6.2. Allium sativum

*Allium sativum* contains various bioactive compounds responsible for its potent antibacterial properties. *A. sativum* oil, rich in diallyl and allyl methyl sulfides, has shown significant effectiveness against *Helicobacter pylori* (*H. pylori*) [67,68,69]. *Allicin*, a key compound, can react with itself to form ajoene, which further enhances *A. sativum*’s antimicrobial action. Allicin’s thiosulfinate group interacts with bacterial enzyme thiol groups, producing mixed disulfides that disrupt biological processes like protein synthesis and DNA replication [70]. These compounds disrupt bacterial cell functions, including membrane integrity, enzyme activity, and metabolic processes, making *A. sativum* a promising natural agent for combating bacterial infections, including multidrug-resistant strains [67].

Research highlights *A. sativum*’s broad-spectrum antibacterial activity, effective against gram-negative and gram-positive bacteria, such as *E. coli*, *Salmonella*, *Shigella*, *S. aureus*, *Pseudomonas aeruginosa*, and *Listeria monocytogenes* [67]. The ethanol extract of *A. sativum* has shown efficacy against multidrug-resistant pathogens, including Mycobacterium tuberculosis and vancomycin-resistant *S. aureus* [67]. Additionally, *A. sativum*’s antibacterial mechanism is attributed to allicin, which disrupts essential metabolic processes by interacting with thiol-containing enzymes such as thioredoxin reductase and RNA polymerase. Also, *A. sativum* extracts inhibit bacterial toxin production and prevent the growth of enterotoxigenic *E. coli* and other intestinal pathogens [34].

Furthermore, the combination of *A. sativum* and ginger has shown a synergistic antibacterial effect, with the most significant inhibition observed against *Mycobacterium tuberculosis* and *E. coli* [71]. These findings underscore *A. sativum*’s potential as a natural, effective antimicrobial agent for treating various bacterial infections. The antibacterial mechanism of *A. sativum* is shown in Figure 2c.

### 6.3. Comparative Perspectives on Geographic and Seasonal Variation

Across regions, both the chemical profile and observed bioactivity of *Allium cepa* and *Allium sativum* vary with cultivar, climate, and processing, which broadens (and sometimes explains) the differences in efficacy reported outside the UAE. For *A. cepa*, Egyptian work in alloxan-diabetic rats showed that onion juice restored glycemic and oxidative-stress markers [72], while a Sudanese clinical study reported acute fasting-glucose reductions after the ingestion of crude red onion—evidence that onions grown and used in different settings can yield clinically meaningful effects in vivo [73]. Complementing this, a Morocco-wide ethnopharmacological review identified *A. cepa* among the most frequently used antidiabetics across multiple provinces, reflecting geographically diverse cultivation and community practices [74]. Cardiovascular findings also differ by material and preparation: pre-clinical data from Pakistan using *A. cepa* bulb oil [75] (, and separate work on onion-peel extracts rich in quercetin [76], demonstrated antithrombotic and myocardial benefits, underscoring that distinct plant parts and local processing can shift the dominant bioactives (e.g., thiosulfinates vs. flavonols). For *A. sativum*, country-level reviews and trials [77,78,79] consistently document antidiabetic actions via sulfur compounds, yet Spain’s studies on aged black garlic show that post-harvest processing (aging) further alters composition—enriching S-allylcysteine and polyphenols—and modulates cardioprotective mechanisms (e.g., NO-mediated vasodilation) [80]. Antibacterial potency also varies by geography, extraction, and strain ecology: reports from Nigeria [81] and Poland [82] show stronger inhibition with ethanolic extracts and synergism with antibiotics against multidrug-resistant pathogens, suggesting that solvent, local chemotype, and clinical context influence outcomes. Together, these examples provide a comparative perspective on how onions and garlic are grown, prepared, and used beyond the UAE—and how regional and seasonal factors (variety selection, climate, harvest time, and processing) can shift phytochemical abundance and, consequently, therapeutic effects.

## 7. Sustainability in Pharmaceutical Sciences

Medicinal plants have long been utilized to treat a wide range of infectious and non-communicable diseases [83,84,85]. They are a rich source of new lead compounds and significant therapeutic agents. Many effective medicinal substances have been created using lead compounds that are naturally sourced or have been extracted straight from plants [86].

For this reason, it is especially crucial to use an interdisciplinary strategy that incorporates phytochemistry, botany, analytical chemistry, traditional and ethnopharmacological expertise, appropriate biological screening techniques, and contemporary drug development methods [87]. New approaches to natural product drug development will reduce obstacles and raise the success rate in the future. New compounds derived from plants, microorganisms, and chemical libraries based on natural products will be used more frequently in drug discovery [88,89,90]. It might play a significant role in developing new medications and addressing issues related to global health [91].

Sustainability in pharmaceutical disciplines has gained significant attention due to the rising need to reduce the environmental and health impacts associated with synthetic chemical drugs. Plant-derived therapeutics can reduce the environmental footprint of drug development by using renewable biomass, generating less hazardous waste, and aligning with green-chemistry principles. Extracts from *Allium* species illustrate this potential: they offer bioactive compounds with clinically relevant activities while enabling eco-friendlier extraction and formulation strategies. Consolidating these approaches advances both therapeutic value and sustainability goals. Plant-based drugs, mainly those derived from medicinal plants like *A. cepa* and *A. sativum*, offer a promising avenue for sustainable therapeutic development by minimizing the synthetic chemical load and encouraging eco-friendly drug discovery and production [91,92].

The pharmaceutical industry habitually depends heavily on synthetic chemicals, which often involve complex chemical processes that generate harmful waste and pose challenges in biodegradability and toxicity [93]. On the other hand, plant-based drugs use bioactive compounds naturally produced by plants, which are generally biodegradable, less toxic, and can be obtained through renewable agricultural practices [65]. This aligns with the sustainability principles outlined earlier in this section.

*Allium* species, particularly *A. sativum* and *A. cepa*, are rich in bioactive phytochemicals such as allyl sulfides, flavonoids, and phenolic compounds, which display a wide spectrum of pharmacological activities, including antimicrobial, anticancer, antioxidant, antihypertensive, and antidiabetic effects [22,24]. These natural compounds have been shown to successfully treat or prevent various chronic diseases, thereby providing safer alternatives to synthetic drugs with fewer side effects and environmental hazards [94].

Furthermore, the use of *Allium* extracts in green synthesis approaches, where plant extracts act as reducing and stabilizing agents in the creation of nanoparticles, illustrates an advanced strategy to develop innovative therapeutics with minimal chemical waste and energy consumption [95]. This eco-friendly synthesis method further enhances the sustainability profile of pharmaceutical development by avoiding the use of toxic chemicals typically employed in conventional nanoparticle synthesis [95].

Moreover, *A. sativum* extracts, which are rich in organosulfur compounds, have exhibited significant antimicrobial activity against multidrug-resistant bacteria, suggesting their potential as alternatives or adjuncts to conventional antibiotics in combating resistant infections [96]. *Allium cepa* extracts also donate antioxidant and anti-inflammatory properties that support their use in managing chronic inflammatory conditions and cardiovascular diseases [97].

Many plants show a significant effect not only on humans but also on other plants [98,99]. Outside their direct therapeutic effects, these plants possess agricultural benefits as natural pesticides, attributable to their insecticidal and antifungal properties, reducing reliance on synthetic agrochemicals and supporting sustainable agriculture [100]. For example, the use of *A. sativum* vermicompost derived from littoral plant waste has been verified to enhance soil quality and support sustainable cultivation practices by reducing reliance on synthetic fertilizers [101].

Likewise, sustainability evaluations of *A. cepa* farming in South Sulawesi and Indonesia reveal that conventional practices involving chemical fertilizers and pesticide carriage environmental and health risks; thus, transitioning to modern, technology-maintained farming systems integrating organic fertilization and improved machinery is recommended to enhance social, economic, and technological sustainability dimensions [102]. Moreover, the conservation and global distribution efforts of *A. cepa* germplasm contribute to agricultural sustainability by conserving the genetic diversity essential for climate adaptation and crop resilience [103].

Interestingly, Suprmanium et al. (2024) demonstrated the role of the exogenous supplementation of biostimulants derived from *A. cepa* peel waste in sustainable biodiesel production by enhancing high-density microalgae cultivation [104].

Together, these studies underscore the importance of adopting combined sustainable cultivation methods such as germplasm conservation, organic amendments, mechanization, and value chain optimization to reduce the environmental footprint of *Allium* crop production while supporting farmer incomes and ensuring long-term resource preservation, which exemplifies a holistic approach to sustainability.

## 8. Enhancement Strategies

### 8.1. Advanced Extraction and Processing Techniques

There are many advanced extraction methodologies for numerous *A. cepa* and *A. sativum* types that demonstrate their applicability to the generation of *A. cepa* extracts with varying chemical compositions [105], such as ultrasound-assisted extraction (UE) and pressurized liquid extraction (PLE). UE is a cost-effective, efficient technique for extracting bioactive compounds from plant cells. It uses ultrasonic waves to disrupt plant cell walls, enhancing solvent penetration and yield, making it ideal for large-scale applications [106,107]. However, PLE is a promising method for obtaining bioactive extracts by using high temperatures and pressures to maintain the solvent in a liquid state above the boiling point.

This method promotes efficient extraction by improving solvent diffusion, reducing viscosity, and decreasing surface tension. Despite its initial investment and energy consumption, PLE offers advantages like improved efficiency, selectivity, and time savings [108]. A recent study compared the two methodologies and determined the yield of multifunctional extracts from *A. sativum* stems, with higher yields of bioactive compounds. UE requires less expensive equipment, lower temperatures, and allows multiple extractions simultaneously. The UE can be applied to different stem *A. sativum* by-products.

These methodologies could be valuable tools for revalorizing *A. sativum* stems and other *A. sativum* by-products in the context of a circular economy. The environmentally friendly nature of these methods, using water as a solvent, ensures safety, sustainability, and low cost, while minimizing *A. sativum* residues and reducing environmental impact [109]. Moreover, microwave-assisted extraction is a quantitative, reproducible, and very promising approach for extracting the phenolic chemicals and anthocyanins of *A. cepa* that produce comparable or even better results with less solvent, time, and cost [110].

### 8.2. Nanotechnology-Based Approaches

Many approaches have been developed to enhance the activity of plant extract-based therapies. Nano drug delivery systems can hold a variety of plant extracts, improving their bioavailability and characteristic properties. The clinical use of plant extracts is restricted due to poor bioavailability, solubility, stability, metabolism, absorption across the intestinal wall, active efflux mechanism, and first-pass metabolic effects [111].

*Allium sativum* oil is known for its high lipophilicity and poor solubility, which limits its bioavailability. However, loading *A. sativum* oil in nanoemulsion has shown stronger anti-inflammatory effects compared to free oil. It reduces gastrointestinal discomfort, enhances absorption, and is more stable than free oil. This encapsulation process increased the absorption and prolonged the shelf life of *A. sativum* oil, making it suitable for adding to various products without compromising its effectiveness [112]. Another study demonstrated the antibacterial potency of nano *A. sativum* against gram-negative bacteria, exhibiting MICs of 12.5–25 μg/mL [113]. Oral nano-delivery systems protect antidiabetic phytocompounds from degradation in the GIT, improve pharmacokinetic and pharmacodynamic profiles, and offer fast action, targeted delivery, sustained release, lower dose, and fewer side effects [114].

Green-synthesized nickel oxide nanoparticles show great promise for new biomedical uses. The co-precipitation approach was utilized to produce nanoparticles from nickel nitrate with *A. cepa* stalk extract as a precursor. These nanoparticles serve as bactericides due to their potential antibacterial activities [115]. Hybrid nanoparticles from *A. cepa* peels proved their potential to inhibit pathogenic microorganisms [116].

## 9. Conclusions

This comprehensive review of *A. cepa* and *A. sativum* within the context of UAE’s local flora and prevalent health issues concludes that these medicinal plants hold significant therapeutic value against diabetes, cardiovascular diseases, and microbial infections. Their diverse biochemical profiles, which include antioxidants, anti-inflammatory agents, and other health-promoting compounds, position them as effective, sustainable alternatives to synthetic medications. These findings support the continued integration of traditional herbal medicine with modern health practices, particularly in regions grappling with the dual challenges of chronic diseases and drug resistance. Furthermore, incorporating these plants within both pharmaceutical and agricultural frameworks demonstrates a comprehensive strategy toward achieving sustainability.

## 10. Future Perspectives

Future research should focus on clinical trials to further establish the pharmacokinetics, safety profiles, and therapeutic indices of these plants. It is also imperative to explore the potential of these plants in combination therapies, possibly enhancing the efficacy of conventional drugs while mitigating side effects. Additionally, genomic and proteomic studies could unravel new bioactive compounds, paving the way for novel drug development.

Safety Validation: various analytical techniques must be used to determine their ingredients, efficacy, safety, and toxicity levels. Pharmacokinetics and ADME studies are crucial for herbal drug development to ensure efficacy, safety, and avoid side effects like kidney damage and infant deaths, and to compete with modern allopathic medicines.

Commercial Production: The herbal drug market faces challenges in quality production, standardization, business law, and regulatory requirements due to poor raw material quality, microbiological contamination, and heavy metal deposition. The FDA prohibits slow-quality and tainted drugs from accessing the global market. To ensure quality, herbal companies should follow WHO recommendations and adhere to drug-controlled authority laws. Herbal medicine contributes to modern healthcare but faces challenges due to a lack of knowledge, technical, and regulatory challenges. Further, the WHO guidelines mandate the continuous process of herbal drug manufacturing, implementing good manufacturing practices to maintain the standard and quality of herbal medicines.

Finally, sustainable cultivation and utilization practices for *A. cepa* and *A. sativum* are needed to ensure the long-term preservation of these valuable medicinal resources.

## 11. Limitations of Existing Studies

In spite of the promising therapeutic potential of *A. cepa* and *A. sativum*, several limitations in existing research delay their translation into typical clinical applications. Addressing these gaps is crucial for advancing evidence-based recommendations and ensuring the reproducibility of therapeutic outcomes.

- Lack of Large-Scale Human Clinical Trials

While in vitro and in vivo preclinical studies dominate the literature, robust human clinical trials remain rare. Most studies on *A. cepa* and *A. sativum* focus on animal models or small-scale human interventions, limiting generalizability. For instance, while *A. sa-tivum* has shown antidiabetic effects in rodent models [43], large randomized controlled trials validating its efficacy in diverse human populations are lacking [46]. Similarly, the cardioprotective effects of *A. cepa* flavonoids (e.g., quercetin) are well documented in vitro, but long-term human studies assessing dose–response relationships and safety are insufficient [53].

b. Standardization Challenges

The variability in bioactive compound composition owing to differences in cultivation, extraction methods, and storage conditions poses a significant barrier. For example, allicin, the primary bioactive compound in *A. sativum*, degrades rapidly, leading to in-consistent concentrations in commercial preparations [57]. Moreover, the antioxidant capacity of *A. cepa* extracts varies widely depending on the onion variety (red vs. yellow) and processing techniques [117]. Furthermore, standardized protocols for extraction, quantification, and stability testing are urgently needed to ensure reproducibility [110].

c. Mechanistic and Pharmacokinetic Gaps

Many studies describe phenomenological effects without clarifying the underlying molecular mechanisms. Key gaps include poor solubility and the rapid metabolism of compounds like quercetin in *A. cepa* [118], and allicin in *A. sativum* [119] limit their therapeutic efficacy.

d. Safety and Regulatory Hurdles

Long-term safety profiles, especially for high-dose or nanoformulated extracts, are understudied [115]. In addition, herbal formulations face challenges in meeting good manufacturing practice (GMP) standards, with inconsistencies in labeling and quality control [120].
